# Synergistic Therapeutic Effects of D-Mannitol–Cerium–Quercetin (Rutin) Coordination Polymer Nanoparticles on Acute Lung Injury

**DOI:** 10.3390/molecules29122819

**Published:** 2024-06-13

**Authors:** Yusheng Zhang, Hong Wang, Ruiying Yang, Ying Zhang, Yao Chen, Cuiping Jiang, Xianyu Li

**Affiliations:** 1 Beijing Key Laboratory of Traditional Chinese Medicine Basic Research on Prevention and Treatment for Major Diseases, Experimental Research Center, China Academy of Chinese Medical Sciences, Beijing 100700, China; 2 School of Traditional Chinese Medicine, Southern Medical University, Guangzhou 510515, China; 3 Traditional Chinese Medicine College, China Pharmaceutical University, Nanjing, 211198, China

**Keywords:** quercetin, rutin, cerium ions, D-mannitol, self-assembly, acute lung injury

## Abstract

Acute lung injury (ALI) remains a significant global health issue, necessitating novel therapeutic interventions. In our latest study, we pioneered the use of D-mannitol–cerium–quercetin/rutin coordination polymer nanoparticles (MCQ/R NPs) as a potential treatment for ALI. The MCQ/R NPs, which integrate rutin and quercetin for their therapeutic potential and D-mannitol for its pulmonary targeting, displayed exceptional efficacy. By utilizing cerium ions for optimal nanoparticle assembly, the MCQ/R NPs demonstrated an average size of less than 160 nm. Impressively, these nanoparticles outperformed conventional treatments in both antioxidative capabilities and biocompatibility. Moreover, our in vivo studies on LPS-induced ALI mice showed a significant reduction in lung tissue inflammation. This groundbreaking research presents MCQ/R NPs as a promising new approach in ALI therapeutics.

## 1. Introduction

Damage to the lungs increases permeability, leading to the exudation of protein-rich fluid that accumulates in the lung interstitium and alveoli [1]. Recent data indicate that ALI has one of the highest morbidity and mortality rates among ICU patients [2]. Treatment strategies for ALI depend on its severity and typically include mechanical ventilation, pharmacotherapy, and respiratory support. In the early stages of ALI, a lung-protective ventilation strategy is commonly recommended, supplemented with medications to reduce inflammation and prevent further pulmonary damage [3], such as corticosteroids, surfactants, antioxidants, non-steroidal drugs, and vasodilators [4,5,6,7,8]. However, the clinical application of some drugs for ALI treatment, particularly certain anti-inflammatory agents and antioxidants, is restricted by their side effects. These side effects include gastrointestinal discomfort, allergic reactions, and other systemic adverse effects, which can lead to poor patient compliance and treatment failure [9]. Additionally, the use of glucocorticoids can lead to complications such as fluid and sodium retention, hypertension, immunosuppression, and hyperglycemia [10].

Traditional Chinese medicine (TCM) offers an integrative approach to treating ALI by enhancing the efficacy of other treatments, holistically adjusting the body’s condition, enhancing self-healing capabilities, and reducing unwanted side effects [11]. Rutin and quercetin, recognized flavonoids of natural origin, are renowned for their exemplary antioxidative and anti-inflammatory properties [12,13]. Recent scientific studies have highlighted their potential pharmacological activity against ALI. For instance, Chen et al. investigated the mechanism by which quercetin protects against sepsis-induced ALI, emphasizing its ability to alleviate pulmonary pathological injuries and counteract oxidative stress through the SIRT1/AMPK pathway [14]. Similarly, Yeh et al. demonstrated that rutin could counteract LPS-induced ALI by inhibiting MAPK-NF-κB activation and increasing antioxidative enzyme activity. Intriguingly, rutin’s efficacy in ameliorating ALI, attributed to its ability to chelate extracellular metal ions, was found to be superior to that of dexamethasone [12]. Furthermore, Basaran et al. suggested that co-administering rutin with other antioxidants, particularly quercetin, could enhance therapeutic outcomes, reduce drug resistance, and attenuate adverse effects [15]. In a study by Yeh and colleagues, the combined use of quercetin and rutin was observed to enhance anti-proliferative activity against the MDA-MB-231 cell line in MTT cell proliferation assays, surpassing the effects of each compound used separately. Additionally, their combined antioxidative capacity was found to be superior to their individual effects [16]. However, the clinical utility of rutin and quercetin is limited by their low oral bioavailability, poor water solubility, rapid metabolism, and enzymatic degradation [14,17,18].

The nanomedicine drug delivery systems offer several advantages, including improved pharmacokinetics and biodistribution, reduced toxicity, enhanced solubility and stability, controlled drug release, and targeted delivery of therapeutic agents. Nanocarriers can be customized by modifying their composition, size, shape, and surface properties. The primary goal of using nanocarriers in drug delivery systems is to treat diseases effectively while minimizing the side effects [19]. One notable advantage of nanomedicine is its ability to deliver drugs directly to affected areas, thus achieving more efficient and effective treatment while simultaneously reducing drug-related side effects [20]. Metal–phenolic networks (MPNs) are a new class of nanomaterials, formed by the self-assembly of metal ions and polyphenols, with bioadhesive properties, favorable biocompatibility, versatile drug loading abilities, and stimuli-responsive characteristics, thus aiding in the diagnosis and treatment of diseases [21]. Considered an emerging class of supramolecular surface modifiers, MPNs show promise in various fields, including drug delivery. For example, a study described a unique core–satellite nanosystem integrated with MPNs, where the “core” consists of a liposome loaded with EDTA, a metal ion chelator [22]. MPN-based drug delivery systems demonstrate excellent stability, adequate drug loading capacity, good biocompatibility, reduced premature release, and remarkable therapeutic effectiveness, thus enabling gradual, multi-stimuli-responsive drug delivery [23]. The ease of preparation, outstanding characteristics, and promising medical applications of MPNs have attracted considerable attention. By coordinating phenolic ligands and metal ions, MPNs demonstrate their potential as multifunctional theranostic nanoplatforms, possessing unique attributes like a rapid preparation process, minimal cytotoxicity, and pH responsiveness [24,25]. The use of metal–organic frameworks and the formation of metal chelates, such as those involving cerium ions, can effectively tackle these challenges [26]. The hydroxyl and carbonyl groups in the structures of quercetin and rutin serve as strong metal chelators [27]. Studies have shown that quercetin–metal chelates, due to their geometric orientation and the presence of metal ion binding sites, demonstrate superior in vitro pharmacokinetics and bioactivity compared to quercetin alone [28]. For instance, Hosseinzadeh et al. developed a delivery system that targets the MDA-MB-231 and A375 cancer cell lines by chelating curcumin and quercetin with cerium ions [26]. Furthermore, cerium oxide has demonstrated potential in preventing and treating ALI by regulating leukocyte recruitment, reducing inflammation and oxidative stress, decreasing collagen deposition, and thus improving pulmonary mechanics [29,30,31]. A recent study showed that administering 0.5 mg/kg of cerium oxide via intraperitoneal injection before exposure to sevoflurane significantly decreased lung injuries in rats, highlighting the potential of cerium oxide in mitigating pulmonary damage [32].

In cases of acute lung injury, there is a notable increase in the expression of macrophages within the lung [33]. To explore the feasibility of efficiently delivering anti-tuberculosis drugs to alveolar macrophages, researchers used mannitol to create microparticles encapsulating highly water-soluble drugs in a specific study. Hegde et al. discovered that, compared to the administration of drugs alone, these mannitol-fabricated microparticles significantly increased drug accumulation within the alveolar macrophages of rats [34]. D-Mannitol, with its distinct 3,4-OH structure, can be recognized by pulmonary surfactant-associated proteins A and D, achieving a degree of lung targeting [35]. Moreover, it significantly enhances the dispersion of nanoparticles in aqueous environments, effectively reducing their propensity to aggregate. Mannitol, as a reliable protective agent, also maintains the structural integrity of nanoparticles during critical processes such as lyophilization and resuspension [36].

In this study, rutin and quercetin synergistically interacted with cerium oxide under optimal conditions to form a metal–polyphenol network. This interaction facilitated the formation of ionic bonds between mannitol and cerium ions. To confirm the formation of the metal–polyphenol network, ultraviolet-visible spectrophotometry (UV), Fourier transform infrared spectroscopy (FTIR), inductively coupled plasma mass spectrometry (ICP-MS), and dynamic light scattering (DLS) analyses were performed. The morphological behavior of the metal–polyphenol network was characterized using transmission electron microscopy (TEM). The antioxidant activities of the coordination polymers were measured through free-radical scavenging assays. Furthermore, the in vitro safety profile was assessed via hemolysis tests. Most importantly, the therapeutic efficacy against acute lung injury was evaluated through a series of experiments involving animal trials, hematoxylin and eosin (H&E) staining, inflammation factor detection, inflammatory cell differential counts, proteomics analysis, and RT-qPCR analysis. This study utilized the lung-targeting properties of mannitol and the ability of nanoparticles to prolong drug blood concentration to investigate the therapeutic advantages of rutin–quercetin metal–polyphenol networks compared to free drugs.

## 2. Results

### 2.1. Size, Zeta Potential, and Encapsulation Efficiency Distribution of MCQ/R NPs

**Figure 1:**
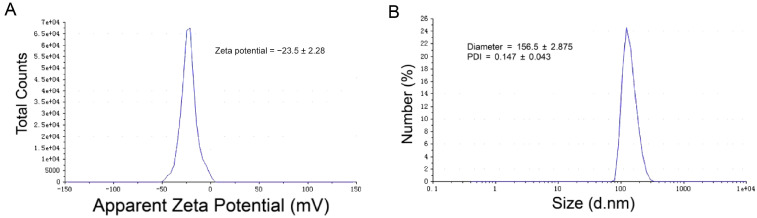
Particle sizes and zeta potential of MCQ/R NPs. (**A**) MCQ/R NPs’ zeta potential was measured with the F13 formulation. (**B**) MCQ/R NPs’ particle size was measured with the F13 formulation.

Based on the single-factor screening results presented in Table 1 showing the composition of NPs, the optimal formulation for the size and zeta potential of MCQ/R NPs was selected. Repeated experiments were conducted according to this formulation. The finalized protocol involved drawing 110 μL of a mixed solution of rutin and quercetin (22.73 mg/mL) and 250 μL of Tris-HCl 8.8 solution into a reaction flask, followed by magnetic stirring at room temperature for five minutes. Subsequently, 31 μL of cerium dioxide solution was added dropwise to the reaction system and allowed to react for another five minutes. Under continuous magnetic stirring, 4 mL of mannitol (10 mg/mL) was added dropwise to the reaction flask and stirred for 24 h. The coordination polymer exhibited satisfactory results in terms of particle size and zeta potential. As shown in Table 2, the results of F13 showed that the particle size of MCQ/R NPs was approximately 156.5 ± 2.875 nm, the zeta potential was −23.5 ± 2.28 mV, and the PDI was 0.147 ± 0.043. Considering these three aspects, the relatively small PDI indicates that MCQ/R NPs formulated under F13 are more stable compared to other formulations (Figure 1).

### 2.2. TEM and ICP-MS Analysis of Coordination Polymers

**Figure 2:**
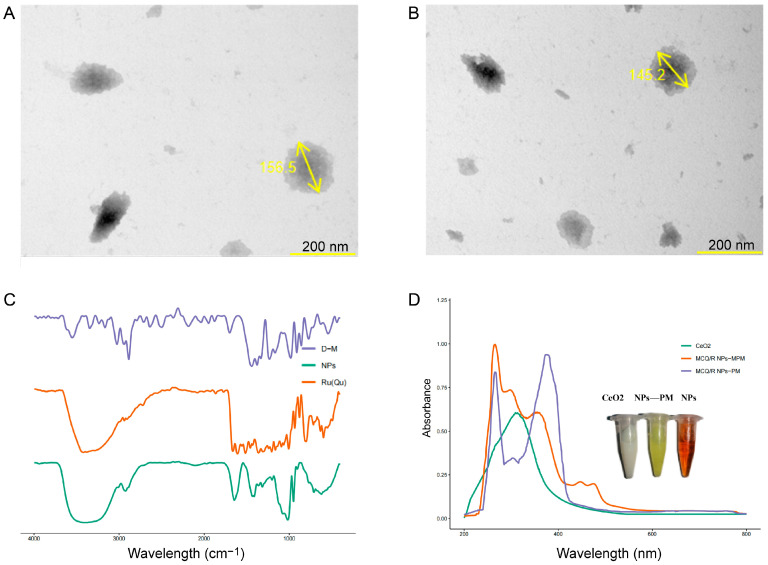
Characterization of coordination polymers. (**A**,**B**) TEM image of MCQ/R NPs; (**C**) the UV–Vis spectra of MCQ/R NPs; (**D**) the FTIR spectra of MCQ/R NPs. For the FTIR spectra, D-M represents a mannitol solution, Ru(Qu) represents a quercetin (rutin) free drug solution, and NPs represent MCQ/R NP nanoformulations. For the UV–Vis spectra, MCQ/R NPs-PM represents the unreacted physical mixture of the MCQ/R NP formulation, and MCQ/R NPs-MPM represents the MCQ/R NP nanoformulations.

As depicted in Figure 2A,B, showing the characterization of coordination polymers, the morphological characteristics of the coordination polymers were observed using transmission electron microscopy (TEM). We identified that the coordination polymers exhibited a square shape with a relatively uniform size. To further determine the cerium content in the coordination polymers, inductively coupled plasma mass spectrometry (ICP-MS) was employed. The results indicated a cerium content of 117.91 mg/kg. The formation process of the coordination polymer of rutin, quercetin, mannitol, and cerium ions in a tris-HCL (pH 8.8) environment was visualized through TEM. Rutin, quercetin, and mannitol acted as ligands, forming coordination bonds with the vacant orbitals of cerium ions, leading to the generation of primary coordination complexes. These primary complexes might further undergo polymerization reactions under specific conditions, resulting in coordination polymers with a uniform size and regular morphology. The TEM images revealed the morphological features of these coordination polymers, showcasing a consistent square structure. This morphological trait underscores the significant orderliness and uniformity in the process of the formation of coordination polymers between rutin, quercetin, mannitol, and cerium ions under specific reaction conditions. The relatively uniform square structure might also reflect the lattice structure and coordination of geometric characteristics within the coordination polymer, suggesting a specific coordination mode during the complexation of rutin, quercetin, mannitol, and cerium ions.

### 2.3. UV–Vis Spectra of MCQ/R NPs

The UV–visible absorption spectra of MCQ/R NPs and their physical mixture solution were recorded between 200 and 800 nm using a UV–visible spectrophotometer (SHIMADZU, UV-2600, Kyoto, Japan). The DMSO solution of rutin–quercetin appeared yellow, and upon mixing with cerium ions, a noticeable color change was observed, turning the MCQ/R NPs to a brownish hue. For the free drug solution, post-reaction with cerium ions, the color transitioned from yellow to brownish. This was hypothesized to be attributed to the successful formation of the coordination polymer. Thus, a series of characterizations were undertaken. As illustrated in Figure 2, compared to the solution of its physical mixture, the spectrum of MCQ/R NPs exhibited a significant shift in the wavelength of maximum absorbance. The UV spectrum of MCQ/R NPs revealed absorption peaks at λ = 356, 302, and 256 nm, while the free drug physical mixture solution displayed peaks at λ = 374, 310, and 256 nm. CeO_2_ showed an absorption peak around λ = 310 nm, and mannitol exhibited no absorption peaks. Given that the chelation site of cerium ions is within the cinnamoyl system (B-ring) of the rutin–quercetin brass structure, if a chelation reaction occurred, there would be a blue shift in the maximum absorption wavelength of the B-ring while the A-ring remained almost unchanged. The results indicated an absorption peak at λ = 374 nm for the free drug physical mixture solution. Upon the addition of cerium ions, the maximum absorbance wavelength for MCQ/R NPs shifted to 356 nm. Meanwhile, the free drug physical mixture solution showed an absorption peak at λ = 256 nm, which remained unchanged for MCQ/R NPs post-cerium ion addition. The free drug physical mixture solution had an absorption peak at λ = 310 nm, which, upon cerium ion addition, shifted to 302 nm for MCQ/R NPs (Figure 2C). These changes in absorption peaks validate the occurrence of chelation and its impact on the structure of rutin–quercetin. Specifically, when cerium ions form a coordination polymer with rutin and quercetin, the maximum absorption wavelength of the B-ring undergoes a blue shift, resulting in MCQ/R NPs with specific UV–Vis spectral characteristics.

### 2.4. Infrared Spectra of MCQ/R NPs

Infrared spectra were recorded for mannitol, the free drug solution of quercetin (rutin), and MCQ/R NPs. The results revealed characteristic absorption peaks of quercetin and mannitol in the infrared spectrum of MCQ/R NPs, albeit with some shifts. The vibrational frequency of the carbonyl group (C=O) in the free drug solution of quercetin (rutin) was located at 1750 cm^−1^. Upon nanoparticle formation, the C=O group exhibited a shift towards a lower wavenumber, settling at 1680 cm^−1^, suggesting the involvement of the quercetin carbonyl group in coordination. Compared to the vibrational frequency of quercetin at 1310 cm^−1^ corresponding to C–OH (at the C-3 position), this peak was absent in the nanoparticles, indicating that this structure also participated in coordination with cerium ions (Figure 2D). The changes in the characteristic absorption peaks of quercetin and rutin after nanoparticle formation indicate their coordination with cerium ions.

### 2.5. Stability Assessment of MCQ/R NPs

**Figure 3:**
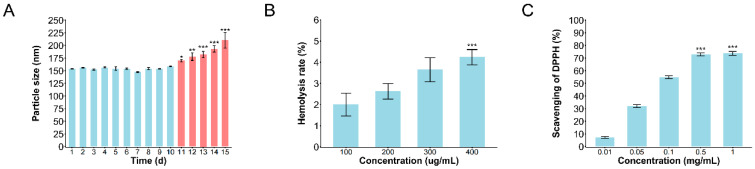
Evaluation of the stability, in vitro safety, and antioxidant activity of MCQ/R NPs. (**A**) Time stability of MCQ/R NPs solution. * *p* < 0.05; ** *p* < 0.01;*** *p* < 0.001. Day 1 was compared with days 11–15. In the bar chart, the red bars represent significant differences compared to Day 1, while the remaining blue bars represent no significant differences compared to Day 1. N = 3. (**B**) The hemolysis rates of MCQ/R NPs at the concentrations of 100, 200, 300, and 400 μg/mL. *** *p* < 0.001 compared with the concentration of 100 μg/mL. n = 6. (**C**) The scavenging rates of MCQ/R NPs at the concentrations of 0.01, 0.05, 0.1, and 0.5–1.0 mg/mL. *** *p* < 0.001 compared with the concentration of 0.01 mg/mL. n = 6.

The stability experiment results revealed that from Day 1 to Day 15, the particle size of MCQ/R NPs did not exhibit any significant increase for a continuous span of 10 days, with an average PDI value of 0.121. Starting from Day 11, there was a noticeable increase in particle size, and between Day 12 and Day 15, in comparison to Day 1, the particle size progressively enlarged, with an average PDI value of 0.313 (Figure 3A). Upon visual inspection of the solution, a gradual formation of flocculent precipitates was observed, indicating that the coordination polymer maintained good stability during the initial ten days post-production.

### 2.6. Hemolysis Assay

As illustrated in Figure 3, we observed that the hemolysis rate increased with the increase in the concentration of the coordination polymer from 100 to 400 μg/mL. Notably, the hemolysis rate for all concentrations of the coordination polymer remained below 5%, suggesting minimal erythrocyte damage by the drug and indicating a low risk of hemolysis (Figure 3B).

### 2.7. Antioxidant Activities of MCQ/R NPs

As depicted in Figure 3, the DPPH radical scavenging activity increased, with the concentration of the MCQ/R NPs solution ranging from 0.01 mg/mL to 1 mg/mL. Upon reaching a concentration of 0.5 mg/mL, the scavenging rate began to stabilize, with only minor increments, indicating the compound’s commendable free-radical scavenging capability (Figure 3C).

### 2.8. Changes in Lung Wet-to-Dry Weight Ratio

Compared to the control group, the model group exhibited a significant increase in the lung wet-to-dry weight ratio. In contrast, all treatment groups demonstrated a marked reduction in this ratio when compared to the model group. Among them, the MCQ/R NPs group showed the most pronounced effect, suggesting that MCQ/R NPs effectively reduced the wet-to-dry weight ratio in the ALI model, thereby alleviating the extent of pulmonary edema (Table 3).

### 2.9. Inflammatory Cell Differential Count

**Figure 4:**
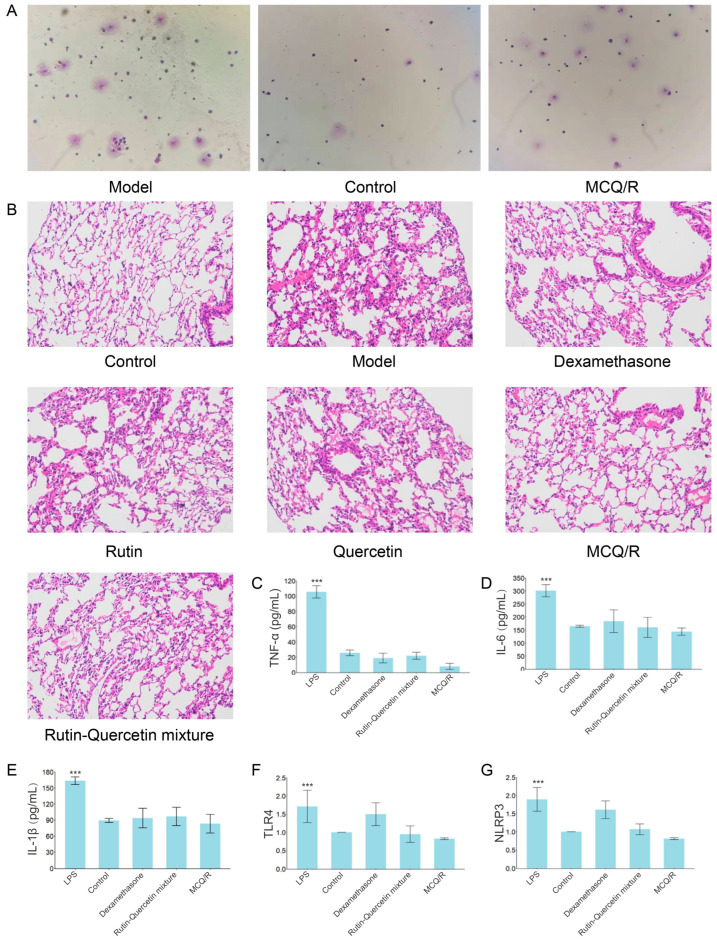
Evaluation of the in vivo safety and therapeutic efficacy of MCQ/R NPs. (**A**) Effect of MCQ/R NPs on the number of inflammatory cells in the BALF of LPS-ALI mice (Giemsa, ×400). (**B**) Effect of MCQ/R NPs on lung histopathology in LPS-ALI mice (HE, ×200). (**C**–**E**) Expression of TNF-α, IL-1β, and IL-6 was detected using ELISA. *** *p* < 0.001 compared with MCQ/R group. N = 6. (**F**,**G**) Expression of TLR4 and NLRP3 mRNA was detected using RT-qPCR. *** *p* < 0.001 compared with MCQ/R group. n = 6.

Compared to the control group, the model group displayed a significant increase in the total cell count and inflammatory cells (neutrophils and macrophages) in the BALF. In contrast, the MCQ/R NPs group showed a marked reduction in inflammatory cells compared to the model group, with comparable cell counts to the control group. This suggests that MCQ/R NPs effectively reduce the number of inflammatory cells in the ALI model, alleviating inflammatory cell infiltration and modulating the inflammatory response associated with ALI (Figure 4A).

### 2.10. Histopathological Examination of Mouse Lung Tissue via Hematoxylin and Eosin (HE) Staining

As depicted in Figure 4B, optical microscopy (×200 magnification) revealed the histological features of the right lung tissue in mice. Compared to the model group, the control group exhibited a normal cellular morphology and overall lung structure, with no evident signs of fibrotic changes, hemorrhage in the alveolar septa, or inflammatory infiltration. In contrast, the LPS-induced model group displayed marked heterogeneity in alveolar size and shape, with some areas even showing a loss of structural integrity. The orderly arrangement was disrupted, and the thickness of the alveolar septa increased, indicating fibrotic alterations. Additionally, hemorrhages in the alveolar septa and signs of inflammatory infiltration were evident, aligning with the typical pathological features of ALI. Upon comparison across experimental groups, improvements or reductions in alveolar size, the thickness of the alveolar septa (indicative of fibrosis degree), the degree of inflammatory infiltration, and pulmonary interstitial edema were observed in the dexamethasone group, rutin group, quercetin group, rutin–quercetin mixed-solution group, and the MCQ/R NPs group relative to the model group. Specifically, these groups exhibited a trend towards normalization of lung tissue structure. When ranking based on significance, the dexamethasone and MCQ/R NPs groups emerged as the most effective in ameliorating the pathological changes. The rutin–quercetin mixed-solution group followed, with the quercetin group trailing and the rutin group showing the least efficacy. In summary, all treatment groups demonstrated significant improvements in alveolar size, the thickness of the alveolar septa, the degree of inflammatory infiltration, and interstitial edema compared to the model group. Notably, the dexamethasone and MCQ/R NPs groups showcased the most pronounced effects, highlighting their potential benefits in alleviating pulmonary fibrosis and inflammation.

### 2.11. Serum Inflammatory Cytokine Levels of IL-6, IL-1β, and TNF-α

Compared to the control group, the model group exhibited a significant elevation in the expression of the serum cytokines IL-6, IL-1β, and TNF-α. The other treatment groups managed to reduce their expression, with the differences being statistically significant. Notably, the MCQ/R NPs group was the most effective in significantly reducing the expression levels of IL-6, IL-1β, and TNF-α (Figure 4C–E). We found that, in the MCQ/R NPs group, the expression levels of IL-6, IL-1β, and TNF-α were lower than those in the control group. We speculate that this could be due to several reasons. Firstly, for the high-dose rutin–quercetin mixture, these combinations have potent anti-inflammatory properties that can significantly inhibit the production of inflammatory cytokines, even beyond normal physiological levels, resulting in lower expression levels than the control group. Secondly, the antioxidant mechanism may further reduce the inflammatory response by decreasing the production of free radicals and the activation of inflammatory signaling pathways, thereby delaying the onset of the LPS-induced cytokine storm. Thirdly, the synergistic effect of rutin and quercetin with nanoparticles enhances the drug’s efficacy, making it more effective than when used alone. Finally, the use of nanoparticles improves drug delivery efficiency, allowing the drug to more effectively reach and act on target cells, significantly suppressing the production of inflammatory cytokines (see Supplementary Materials).

### 2.12. Expression of TLR4 and NLRP3 mRNA in Lung Tissue

As illustrated in Figure 4, when compared to the control group, the model group displayed a significant upregulation in the expression of TLR4 and NLRP3 mRNA (*p* < 0.01). In contrast to the model group, all treatment groups exhibited a decrease in TLR4 and NLRP3 mRNA expression. Notably, the MCQ/R NPs group demonstrated a significant reduction in TLR4 and NLRP3 mRNA expression when compared to both the model group and the free drug group (Figure 4F,G).

### 2.13. Proteomics Analysis

**Figure 5:**
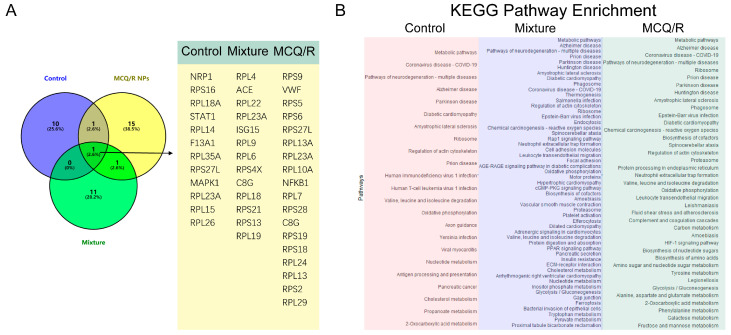
Proteomics analysis. (**A**) In terms of the KEGG enrichment outcomes, a Venn diagram depicting the proteins enriched in the Coronavirus disease–COVID-19 pathway was constructed to compare the differences between the model group and the control group, the model group and the rutin–quercetin mixture group, as well as the model group and the MCQ/R group, along with the corresponding enriched genes in the Coronavirus disease–COVID-19 pathway. (**B**) In the model group, 196 differentially expressed proteins were subjected to KEGG pathway analysis compared to the control group. Additionally, 290 differentially expressed proteins were analyzed in comparison to the rutin–quercetin mixture group, and 224 differentially expressed proteins were compared to the MCQ/R group.

Mass spectrometry and proteomics data retrieval identified 2828 proteins. A statistical analysis was performed of the model group versus the control group, the model group versus the rutin–quercetin mixture group, and the model group versus the MCQ/R group, with a criterion of fold changes > 1.5 (*p* < 0.05). The comparative proteomic analysis revealed a set of differentially expressed proteins, with significant implications. Specifically, between the model and control groups, 196 proteins exhibited pronounced differences; when the model group was compared with the rutin–quercetin mixture group, this number escalated to 290 proteins; and the comparison between the model and MCQ/R group identified 224 proteins with notable variances. The differential proteins from each group were subjected to KEGG pathway enrichment analysis. Our findings revealed that all three groups showed significant enrichment in the coronavirus disease–COVID-19 pathway, which aligns with our construction and treatment of the ALI model. Further analysis of the Coronavirus disease–COVID-19 pathway indicated that the MCQ/R group showed the enrichment of more targets compared to the rutin–quercetin mixture group. Moreover, the total number of enriched pathways in the MCQ/R group was less than that in the rutin–quercetin mixture group. This suggests that MCQ/R, in comparison with the rutin–quercetin mixture, can modulate the treatment of ALI more effectively by targeting a greater number of key regulatory points in a more focused manner (Figure 5A,B).

## 3. Discussion

To effectively deliver rutin and quercetin polyphenols, coordination polymers were constructed using a metal–phenolic network approach within the rutin and quercetin molecules. Cerium dioxide was used as the metal source. Mannitol was added to prevent the aggregation of the coordination polymers, control particle size, and possibly facilitate targeted delivery. The coordination bonds between rutin and quercetin with cerium ions were characterized by FTIR and UV spectroscopy, suggesting interactions between hydroxyl and carbonyl groups and cerium ions, likely resulting in the formation of coordination polymer nanoparticles. Screening results revealed a favorable nanoparticle size and zeta potential. Their stability was highlighted by insignificant variations in size and potential, likely due to the presence of mannitol in the suspension. Since safety is essential for clinical application, the biocompatibility of the coordination polymers was assessed using hemolysis tests. Although hemolysis increased with higher concentrations of the coordination polymer, all samples showed hemolysis rates below 5%, making them suitable for clinical applications. Given the pivotal role of oxidative stress in ALI progression, quenching excessive ROS is a promising therapeutic strategy. Due to their catechol moieties, polyphenols are generally recognized for their antioxidative potential. This hypothesis was validated by assessing the metal–phenolic network’s scavenging activity against DPPH radicals. As the concentration of MCQ/R NPs solution increased, DPPH radical scavenging stabilized, and at 0.5 mg/mL, the scavenging rate exceeded 70%. Animal model studies revealed that MCQ/R NPs attenuated the morphological changes in rat lung tissues induced by lipopolysaccharide (LPS). Optical microscopy revealed significant differences in the lung tissue sections of model mice, characterized by uneven alveolar dimensions, compromised structural integrity, thickened alveolar septa, fibrotic changes, hemorrhage, and inflammatory infiltration. In stark contrast, mice treated with dexamethasone, rutin, quercetin, a mixture of rutin and quercetin, and MCQ/R NPs showed varying degrees of damage alleviation, with lung tissue structures approaching normalcy. Notably, compared to free drugs and the positive control, MCQ/R NPs showed superior efficacy in downregulating TLR4 and NLRP3 mRNA expressions, highlighting significant potential in improving the prognosis of acute lung injury. Additionally, differentially expressed proteins from each group were analyzed using KEGG pathway enrichment. The study found significant enrichment in the coronavirus disease–COVID-19 pathway in all three groups, aligning with our ALI model construction and treatment. Further analysis of the coronavirus disease–COVID-19 pathway indicated that the MCQ/R group had more target enrichment compared to the rutin–quercetin mixture group. Moreover, the total number of enriched pathways in the MCQ/R group was lower than in the rutin–quercetin mixture group. This suggests that MCQ/R, compared to the rutin–quercetin mixture, can modulate ALI treatment more effectively by targeting more key regulatory points in a focused manner.

## 4. Methods, Materials, and Animals

### 4.1. Materials and Animals

Rutin (purity ≥ 98%) was purchased from Chengdu Efa Biotech Co., Ltd. (Chengdu, China). Quercetin (purity ≥ 98%) was sourced from Chengdu Efa Biotech Co., Ltd. (Chengdu, China). D-Mannitol was obtained from Beijing Solarbio Science & Technology Co., Ltd. (Beijing, China). Tris-HCl (pH 8.8) was acquired from Shanghai Biyuntian Biotech Co., Ltd. (Shanghai, China). Dialysis bags (3.5 KDa) and ultrafiltration tubes (3 KDa) were provided by Guangzhou Yuecan Laboratory Instrument Co., Ltd. (Guangzhou, China). Reactive oxygen species (ROS) assay kit was procured from Shanghai Biyuntian Co., Ltd. (Shanghai, China). Wright-Giemsa stain was bought from Sigma-Aldrich (St. Louis, MI, USA). Cerium dioxide solution (CeO_2_) was obtained from Shanghai Aladdin Biochemical Technology Co., Ltd. (Shanghai, China).

BALB/c mice, SPF grade, aged 6–8 weeks and weighing 20–25 g, were supplied by the Animal Experimental Center of Southern Medical University (Guangzhou, China). The animals were housed at a temperature of (24 ± 2) °C with a relative humidity of 50–60%. They had ad libitum access to food and water.

### 4.2. Synthesis and Characterization of MCQ/R NPs

**Figure 6:**
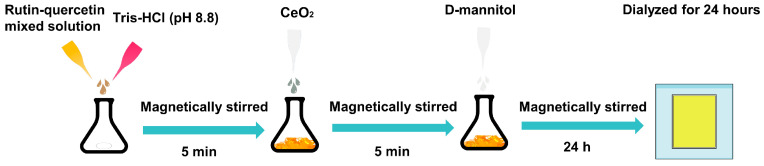
Preparation procedure of MCQ/R NPs. An appropriate volume of a mixed solution of rutin and quercetin is added to a reaction flask containing Tris-HCl (pH 8.8). The mixture is stirred magnetically at room temperature for five minutes. Subsequently, an optimal volume of CeO_2_ is reacted with for an additional five minutes. Finally, under continuous magnetic stirring, D-mannitol is titrated into the reaction mixture and stirred for 24 h.

The synthesis of MCQ/R NPs nanoparticles proceeded as follows: An appropriate volume of a mixed solution of rutin and quercetin was combined with an adequate amount of Tris-HCl (pH 8.8) in a reaction flask and stirred magnetically at room temperature for five minutes. Subsequently, an optimal volume of cerium dioxide solution was introduced into the system and allowed to react for an additional five minutes. Finally, under continuous magnetic stirring, D-mannitol was incrementally added dropwise to the reaction mixture and stirred for 24 h (Figure 6). The resulting solution was then dialyzed for 24 h and stored in ambient conditions. UV–Vis, FTIR, ICP-MS, and TEM were used for a comprehensive characterization. Malvern Zetasizer Nano ZS facilitated precise measurements of particle diameter, zeta potential, and PDI, offering intricate insights into nanoparticle morphology, structure, and cerium ion content.

### 4.3. Characterization of Coordination Polymers

#### 4.3.1. TEM and ICP-MS Analysis

For TEM analysis, suspensions of each coordination polymer were deposited onto copper grids coated with carbon film, stained with a 2% (*w*/*v*) tungstophosphoric acid solution, and air-dried at room temperature. The morphological behaviors of each sample were visualized using a transmission electron microscope operating at 120 kV (Hitachi, Tokyo, Japan). For ICP-MS examination, coordination polymer samples were analyzed using an Agilent 7700 ICP-MS instrument with suitably pure and concentrated sample solutions. The ICP-MS instrument was configured with specific parameters: RF power at 1.55 kW, RF matching at 1.80 V, auxiliary gas flow at 1.50 L/min, carrier gas flow at 0.71 L/min, and makeup gas flow at 0.48 L/min. Sample solutions were introduced to the instrument over a 45 s uptake period, allowing the instrument to initiate after a 30 s stabilization interval. Data generated by the ICP-MS instrument were collected and analyzed with an integration time set at 0.90 s per mass, providing insights into the mass and composition of the coordination polymer samples.

#### 4.3.2. UV–vis Spectrophotometry

The structural composition of MCQ/R NPs was determined using ultraviolet (UV) spectroscopy. The procedure involved the sequential sampling and testing of appropriate volumes of the MCQ/R NP solution and a physical mixture solution of the coordinating polymer [37].

#### 4.3.3. FTIR Spectroscopy

Fourier-transform infrared (FTIR) spectroscopy was employed to determine the structural composition of MCQ/R NPs. The method involved evaporating and drying an adequate volume of the MCQ/R NP solution to produce a powder. An appropriate amount of physically mixed coordinating polymer powder was then sampled, followed by sequential testing of the aforementioned two powders and D-mannitol powder [37].

### 4.4. Stability Test of MCQ/R NPs

The prepared MCQ/R NPs solution was stored at room temperature. Its stability was assessed through continuous visual observations over several days and a particle size analysis was conducted using a laser particle size analyzer.

### 4.5. Radical Scavenging Ability Experiment

We accurately weighed 5.9148 mg of DPPH using an analytical balance and transferred it into a brown glass bottle. We then dissolved it in anhydrous ethanol and diluted it to a volume of 100 mL, resulting in a DPPH working solution of 150 μmol/L. A volume of 180 μL of this working solution was mixed with 20 μL of sample solutions at concentrations of 0.01, 0.05, 0.1, 0.5, and 1 mg/mL. The mixtures were gently agitated to ensure homogeneity. For the positive control, distilled water (dH_2_O) was used instead of the sample solution. Meanwhile, for the negative control, anhydrous ethanol was used instead of DPPH. After incubation in the dark for 30 min, absorbance readings were taken at 517 nm for all groups using an enzyme-linked immunosorbent assay (ELISA) plate reader [37]. The DPPH radical scavenging rate was calculated using the following equation:

$$\mathrm{DPPH}\;\mathrm{scavenging}\;\mathrm{rate}\;(\%)=(1-\frac{Asample-Anegative\;control}{Apositive\;control})\;\times \;100\%$$

### 4.6. Hemolysis Test

Cells were repeatedly washed with PBS (pH 7.4) and then centrifuged at 2500 rpm for 10 min. The pellet was resuspended in PBS to achieve a 2% (*v*/*v*) red blood cell suspension. The sedimented red blood cells were washed 3–4 times with PBS (pH 7.4) until the supernatant was no longer red. Different concentrations of the MCQ/R NPs solution (100, 200, 300, and 400 µg/mL) were mixed with an equal volume of the 2% red blood cell suspension and incubated at 37 °C on a shaker at 65 rpm for 3 h. After incubation, the mixtures were centrifuged at 3000 rpm for 10 min, and 200 μL of the supernatant was collected into a 96-well plate. The absorbance of the released hemoglobin in the supernatant was measured at 540 nm. Red blood cell suspensions incubated with either deionized water or physiological saline served as positive and negative controls, respectively [37]. The hemolysis rate was calculated using the following formula, where the original sample was replaced by an equivalent volume of PBS (pH 7.4) for incubation with the red blood cell suspension.

$$\mathrm{Hemolysis}\;\mathrm{rate}\;(\%)=(1-\frac{Asample-Aoriginal\;sample}{Apositive\;control-Anegative\;control})\;\times \;100\%$$

### 4.7. Animal Grouping, Administration, Modeling, and Sample Collection

Following 5 days of acclimatization, mice were weighed, ranked by weight, and stratified. Grouping was randomized using a random number table. Except for the blank group, acute lung injury models were induced via the intraperitoneal injection of a 5 mg/kg LPS saline solution. Twelve hours post-modeling, and based on the literature conversion, mice were administered equivalent doses. The groups consisted of blank, LPS (5 mg/kg), dexamethasone (5 mg/kg), rutin (100 mg/kg), quercetin (100 mg/kg), a mixed solution of rutin and quercetin (100 mg/kg each), and MCQ/R NPs (50 mg/kg). Each group contained 10 mice, with intraperitoneal injections administered every 12 h for 5 consecutive days. The blank and model groups received equivalent volumes of saline. Blood was drawn from the orbit 12 h after the final dose, and serum was separated for storage. From each group, three mice underwent bronchoalveolar lavage by exposing the trachea, inserting a soft tube, and washing with pre-cooled PBS (pH 7.4), ensuring an 85% retrieval rate. The BALF was centrifuged at 3000 rpm (centrifuge radius: 10 cm) for 10 min at 4 °C. A total of 0.8 mL of the supernatant was aliquoted for total and inflammatory cell counts. After BALF collection, lung tissues were washed with PBS (pH 7.4). The right lung was fixed in 4% neutral formalin, the left lung was stored at −80 °C, and other mice were similarly processed to compute W/D values. Heart, liver, spleen, and kidney tissues were also collected and stored at −80 °C.

### 4.8. Pulmonary Tissue Hematoxylin and Eosin (H&E) Staining

Mouse left lung tissues were thoroughly rinsed with water for several hours. The tissues were then dehydrated through a graded ethanol series of 70%, 80%, and 90%. This was followed by a 15 min incubation in an equal mixture of pure ethanol and xylene, and subsequent xylene treatments for 15 min each until transparency was achieved. The tissues were then immersed in a 50:50 mixture of xylene and paraffin for 15 min, followed by sequential immersion in paraffin I and paraffin II, each for 50–60 min. After embedding in paraffin, sections were cut. The paraffin sections were baked, dewaxed, and rehydrated. The rehydrated sections were stained with hematoxylin solution for 3 min, differentiated with hydrochloric acid ethanol for 15 s, briefly rinsed with water, blued for 15 s, and then rinsed again. Eosin staining was applied for 3 min, followed by a thorough rinsing. The sections were then dehydrated, cleared, mounted, and examined under a microscope.

### 4.9. Serum Levels of TNF-α, IL-1β, and IL-6 Were Measured in Mice

Blood was drawn from the orbital plexus and allowed to clot at room temperature for 2 h. The samples were then centrifuged at 4 °C at 3000 rpm for 10 min. The supernatant, representing the serum, was collected. Assays were performed using ELISA according to the manufacturer’s protocol.

### 4.10. Quantitative Real-Time PCR (qRT-PCR) Was Employed to Assess mRNA Expression Levels

Total RNA was extracted from lung tissues utilizing an RNA extraction kit, followed by the determination of RNA concentration. For the qRT-PCR analysis, we used the SYBR Premix Ex Taq™ II (Tli RNase H Plus) with the Archimed-X6 real-time PCR system. The forward (F) and reverse (R) primers used for the qRT-PCR are listed in Table 4. 

### 4.11. Proteomics Analysis

Protein concentration was measured at 280 nm using a NanoDrop spectrophotometer (Thermo, Waltham, MA, USA) with an extinction coefficient of 1.1 AU. The Filter-Aided Sample Preparation (FASP) protocol was used for proteolytic digestion of proteins to remove detergents from the lysate, employing centrifugal units with a molecular weight cutoff of 30,000. This involved adding 200 μL of 8 M urea in 0.1 M Tris/HCl, pH 8.5 (UA buffer), to YM-30 Microcon filter units (Millipore, Burlington, MA, USA) containing CM protein concentrates and centrifuging at 14,000× *g* for 15 min at 20 °C, repeated twice. A total of 50 μL of 0.05 M iodoacetamide in 8 M urea was added to the filters, incubated for 20 min in the dark, and washed twice with 100 μL of 8 M UA buffer and three times with 100 μL of 50 mM NH_4_HCO_3_. A total of 100 μL of 50 mM NH_4_HCO_3_ containing trypsin (Promega, Madison, WI, USA) was added to each filter at a protein-to-enzyme ratio of 100:1. Samples were incubated overnight at 37 °C, and the digested peptides were collected by centrifuging at 14,000× *g* for 15 min at 20 °C. The peptides were then analyzed online using an Orbitrap Fusion Lumos Tribrid mass spectrometer (Thermo Fisher Scientific, Waltham, MA, USA) coupled with a nano-flow HPLC (EASY-nLC 1000 system, Thermo Fisher Scientific, USA) and a self-packed chromatography column with Ultimate XB-C18 3 μm resin (Welch Materials, West Haven, CT, USA). The peptide mixtures were separated on a C18 reversed-phase column (10 cm length, 75 μm inner diameter) using a 75 min linear gradient of 3–100% buffer B (99.5% acetonitrile, 0.5% formic acid) in buffer A (99.5% water, 0.5% formic acid) at a flow rate of 350 nL/min. The total LC-MS/MS run time, including loading and cleaning steps, was approximately 90 min. The electrospray voltage was set to 2.0 kV. During data-dependent MS/MS acquisition, the dynamic exclusion time was set to 18 s. In the MS1 stage, the resolution was set to 70,000 and the AGC target to 3 × 10^6^, with a maximum injection time of 20 ms. In the MS2 stage, the resolution was 17,500, the AGC target was 1 × 10^6^, and the maximum injection time was 60 ms. The scan range was set to 300–1400 *m*/*z*, with the top 20 strongest precursor ions selected for MS/MS analysis. The raw data were subjected to database searching, and significant proteins were selected using R Studio software, with *p*-values of less than 0.05 set as the threshold for the statistical analysis of differentially expressed proteins between the control and model groups and the control and high-dose groups. The PPI networks of the differential proteins of the two groups were integrated and analyzed.

### 4.12. Statistical Analysis

Image J (Bio-Rad, Hercules, CA, USA) was used for the quantitative analysis of target bands on Western blots. Histograms and statistical analyses were performed using GraphPad Prism (GraphPad Prism 9.0, San Diego, CA, USA). The results are presented as the mean ± standard deviation (SD). Analysis of variance (ANOVA) followed by Tukey’s post-hoc test was used to compare means among multiple groups. Significance levels were set at *p* < 0.05, *p* < 0.01, and *p* < 0.001.

## 5. Conclusions

In this study, rutin and quercetin acted both as therapeutic agents and as self-carrier materials, interacting with cerium ions as the connecting points and mannitol as a potential targeting delivery carrier, ultimately self-assembling into MCQ/R nanoparticles. These nanoparticles demonstrated a clear therapeutic effect in the treatment of acute lung injury while maintaining good biocompatibility.

## Figures and Tables

**Table 1:** Composition of NPs.

S/N	Volume of CeO_2_ (μL)	Volume of Medicines (μL)	Volume of Tris-Hcl 8.8 (μL)	Volume of D-Mannitol (mg/mL)
F1	21	110	200	4 (20 mg/mL)
F2	31	110	200	4 (20 mg/mL)
F3	41	110	200	4 (20 mg/mL)
F4	31	65	200	4 (20 mg/mL)
F5	31	110	200	4 (20 mg/mL)
F6	31	175	200	4 (20 mg/mL)
F7	31	110	150	4 (20 mg/mL)
F8	31	110	200	4 (20 mg/mL)
F9	31	110	250	4 (20 mg/mL)
F10	31	110	250	4 (10 mg/mL)
F11	31	110	250	4 (20 mg/mL)
F12	31	110	250	4 (40 mg/mL)
F13	31	110	250	4 (10 mg/mL)

**Table 2:** Diameter, zeta potential, and polydispersity index of the NPs (n = 3).

S/N	Diameter (nm)	Polydispersity Index	Zeta Potential (mV)
F1	259.3 ± 3.13	0.273 ± 0.02	−16.5 ± 1.31
F2	187.4 ± 4.93	0.170 ± 0.07	−13.9 ± 0.80
F3	321.2 ± 18.47	0.260 ± 0.03	−30.8 ± 2.65
F4	141.9 ± 1.17	0.175 ± 0.03	−24.7 ± 2.50
F5	163.9 ± 1.66	0.210 ± 0.02	−24.8 ± 1.54
F6	288.6 ± 4.29	0.302 ± 0.05	−25.6 ± 0.85
F7	156.2 ± 1.25	0.198 ± 0.81	−17.9 ± 3.41
F8	170.3 ± 3.68	0.265 ± 0.94	−22.6 ± 2.11
F9	138.5 ± 4.51	0.147 ± 0.65	−23.5 ± 1.60
F10	138.5 ± 4.51	0.147 ± 0.65	−17.9 ± 3.41
F11	170.3 ± 3.68	0.265 ± 0.94	−22.6 ± 2.10
F12	156.2 ± 1.25	0.198 ± 0.81	−23.5 ± 1.61
F13	156.5 ± 2.875	0.147 ± 0.043	−23.5 ± 2.28

**Table 3:** Effect of MCQ/R on lung moisture–dry weight ratio in LPS-ALI mice (n = 3) * *p* < 0.05.

Group	W/D
Control	5.647 *
LPS	6.659
Dexamethasone	5.716 *
MCQ/R	5.591 *
Rutin	5.847 *
Quercetin	5.601 *
Rutin–Quercetin mixture	5.767 *

**Table 4:** Sequences of the primers.

Gene	Forward Primer (5′–3′)	Reverse Primer (5′–3′)
GAPDH	CATCACTGCCACCCAGAAGACTG	ATGCCAGTGAGCTTCCCGTTCAG
TLR4	AGCTTCTCCAATTTTTCAGAACTTC	TGAGAGGTGGTGTAAGCCATGC
NLRP3	ACCTCAACAGTCGCTACACG	ATGGTTTTCCCGATGCC

## Data Availability

The original contributions presented in the study are included in the article, further inquiries can be directed to the corresponding authors.

## Supplementary Materials

The following supporting information can be downloaded at: https://www.mdpi.com/article/10.3390/molecules29122819/s1.

## References

[1] Tay M.Z., Poh C.M., Renia L., MacAry P.A., Ng L. (2020). The trinity of COVID-19: Immunity, inflammation and intervention. Nat. Rev. Immunol..

[2] Li L.S., Yang Y., Liu X.L., Zhang C.R., Ye Q.Y., Hou W.J., Zhao Y.H., Xiao G.P., Li X.N., Li Y.H. (2017). Pathogenic role of leukotriene b4 in pulmonary microvascular endothelial cell hyper- permeability induced by one lung ventilation in rabbits. Nan Fang Yi Ke Da Xue Xue Bao.

[3] Mokra D. (2020). Acute lung injury—From pathophysiology to treatment. Physiol. Res..

[4] He S., Wu L., Sun H., Wu D., Wang C., Ren X., Shao Q., York P., Tong J., Zhu J. (2022). Antioxidant biodegradable covalent cyclodextrin frameworks as particulate carriers for inhalation therapy against acute lung injury. ACS Appl. Mater. Interfaces.

[5] Tongyoo S., Permpikul C., Mongkolpun W., Vattanavanit V., Udompanturak S., Kocak M., Meduri G.U. (2016). Hydrocortisone treatment in early sepsis-associated acute respiratory distress syndrome: Results of a randomized controlled trial. Crit. Care.

[6] Raghavendran K., Willson D., Notter R.H. (2011). Surfactant therapy for acute lung injury and acute respiratory distress syndrome. Crit. Care Clin..

[7] Kim S.K., Rho S.J., Kim S.H., Kim S.Y., Song S.H., Yoo J.Y., Kim C.H., Lee S.H. (2019). Protective effects of diphenyleneiodonium, an nadph oxidase inhibitor, on lipopolysaccharide-induced acute lung injury. Clin. Exp. Pharmacol. Physiol..

[8] Levitt J.E., Matthay M.A. (2012). Clinical review: Early treatment of acute lung injury--paradigm shift toward prevention and treatment prior to respiratory failure. Crit. Care.

[9] Liu Y., Zhou S., Xiang D., Ju L., Shen D., Wang X., Wang Y. (2021). Friend or foe? The roles of antioxidants in acute lung injury. Antioxidants.

[10] Roberts A., James J., Dhatariya K. (2018). Management of hyperglycaemia and steroid (glucocorticoid) therapy: A guideline from the joint british diabetes societies (jbds) for inpatient care group. Diabet. Med..

[11] Kang X., Jin D., Jiang L., Zhang Y., Zhang Y., An X., Duan L., Yang C., Zhou R., Duan Y. (2022). Efficacy and mechanisms of traditional chinese medicine for covid-19: A systematic review. Chin. Med..

[12] Yeh C.H., Yang J.J., Yang M.L., Li Y.C., Kuan Y.H. (2014). Rutin decreases lipopolysaccharide-induced acute lung injury via inhibition of oxidative stress and the mapk-nf-kappab pathway. Free Radic. Biol. Med..

[13] Chen Y.-B., Zhang Y.-B., Wang Y.-L., Kaur P., Yang B.-G., Zhu Y., Ye L., Cui Y.-L. (2022). A novel inhalable quercetin-alginate nanogel as a promising therapy for acute lung injury. J. Nanobiotechnol..

[14] Chen L.-L., Song C., Zhang Y., Li Y., Zhao Y.-H., Lin F.-Y., Han D.-D., Dai M.-H., Li W., Pan P.-H. (2022). Quercetin protects against lps-induced lung injury in mice via sirt1-mediated suppression of pkm2 nuclear accumulation. Eur. J. Pharmacol..

[15] Satari A., Ghasemi S., Habtemariam S., Asgharian S., Lorigooini Z. (2021). Rutin: A flavonoid as an effective sensitizer for anticancer therapy; Insights into multifaceted mechanisms and applicability for combination therapy. Evid.-Based Complement. Altern. Med..

[16] Basaran E., Ozturk A.A., Senel B., Demirel M., Sarica S. (2022). Quercetin, rutin, and quercetin-rutin incorporated hydroxypropyl beta-cyclodextrin inclusion complexes. Eur. J. Pharm. Sci..

[17] Tian C., Shao Y., Jin Z., Liang Y., Li C., Qu C., Sun S., Cui C., Liu M. (2022). The protective effect of rutin against lipopolysaccharide induced acute lung injury in mice based on the pharmacokinetic and pharmacodynamic combination model. J. Pharm. Biomed. Anal..

[18] Chen W.-Y., Huang Y.-C., Yang M.-L., Lee C.-Y., Chen C.-J., Yeh C.-H., Pan P.-H., Horng C.-T., Kuo W.-H., Kuan Y.-H. (2014). Protective effect of rutin on lps-induced acute lung injury via down-regulation of mip-2 expression and mmp-9 activation through inhibition of akt phosphorylation. Int. Immunopharmacol..

[19] Din F.U., Aman W., Ullah I., Qureshi O.S., Mustapha O., Shafique S., Zeb A. (2017). Effective use of nanocarriers as drug delivery systems for the treatment of selected tumors. Int. J. Nanomed..

[20] Ghasemiyeh P., Mohammadi-Samani S. (2020). Potential of nanoparticles as permeation enhancers and targeted delivery options for skin: Advantages and disadvantages. Drug Des. Dev. Ther..

[21] Liu P., Shi X., Zhong S., Peng Y., Qi Y., Ding J., Zhou W. (2021). Metal-phenolic networks for cancer theranostics. Biomater. Sci..

[22] Gao Y., Yang S.C., Zhu M.H., Zhu X.D., Luan X., Liu X.L., Lai X., Yuan Y., Lu Q., Sun P. (2021). Metal phenolic network-integrated multistage nanosystem for enhanced drug delivery to solid tumors. Small.

[23] Yi X., Zeng W., Wang C., Chen Y., Zheng L., Zhu X., Ke Y., He X., Kuang Y., Huang Q. (2022). A step-by-step multiple stimuli-responsive metal-phenolic network prodrug nanoparticles for chemotherapy. Nano Res..

[24] Wang H., Wang D., Yu J., Zhang Y., Zhou Y. (2022). Applications of metal-phenolic networks in nanomedicine: A review. Biomater. Sci..

[25] Zhang Z., Xie L., Ju Y., Dai Y. (2021). Recent advances in metal-phenolic networks for cancer theranostics. Small.

[26] Hosseinzadeh R., Khorsandi K., Esfahani H.S., Habibi M., Hosseinzadeh G. (2021). Preparation of cerium-curcumin and cerium-quercetin complexes and their leds irradiation assisted anticancer effects on mda-mb-231 and a375 cancer cell lines. Photodiagnosis Photodyn. Ther..

[27] Leopoldini M., Russo N., Chiodo S., Toscano M. (2006). Iron chelation by the powerful antioxidant flavonoid quercetin. J. Agric. Food Chem..

[28] Dolatabadi J.E. (2011). Molecular aspects on the interaction of quercetin and its metal complexes with dna. Int. J. Biol. Macromol..

[29] Niemiec S.M., Hilton S.A., Wallbank A., Louiselle A.E., Elajaili H., Hu J., Singh S., Seal S., Nozik E., Smith B. (2022). Lung function improves after delayed treatment with cnp-mir146a following acute lung injury. Nanomed. Nanotechnol. Biol. Med..

[30] Niemiec S.M., Hilton S.A., Wallbank A., Azeltine M., Louiselle A.E., Elajaili H., Allawzi A., Xu J., Mattson C., Dewberry L.C. (2021). Cerium oxide nanoparticle delivery of microrna-146a for local treatment of acute lung injury. Nanomed. Nanotechnol. Biol. Med..

[31] Ozdemirkan A., Kucuk A., Gunes I., Arslan M., Tuncay A., Sivgin V., Sezen S.C., Boyunaga H. (2021). The effect of cerium oxide on lung injury following lower extremity ischemia-reperfusion injury in rats under desflurane anesthesia. Saudi Med. J..

[32] Tuncay A., Sivgin V., Ozdemirkan A., Sezen S.C., Boyunaga H., Kucuk A., Gunes I., Arslan M. (2020). The effect of cerium oxide on lung tissue in lower extremity ischemia reperfusion injury in sevoflurane administered rats. Int. J. Nanomed..

[33] Cheng P., Li S., Chen H. (2021). Macrophages in lung injury, repair, and fibrosis. Cells.

[34] Maeda R., Ito T., Tagami T., Takii T., Ozeki T. (2019). Development of dried emulsion/mannitol composite microparticles through a unique spray nozzle for efficient delivery of hydrophilic anti-tuberculosis drug against alveolar macrophages. Biol. Pharm. Bull..

[35] Su S.H., Chen H.I., Jen C.J. (2005). Exercise enhances surfactant-mediated phagocytosis in bronchoalveolar macrophages. Chin. J. Physiol..

[36] Voci S., Gagliardi A., Salvatici M.C., Fresta M., Cosco D. (2022). Influence of the dispersion medium and cryoprotectants on the physico-chemical features of gliadin- and zein-based nanoparticles. Pharmaceutics.

[37] Tang Q., Yi Y., Chen Y., Zhuang Z., Wang F., Zhang L., Wei S., Zhang Y., Wang Y., Liu L. (2022). A green and highly efficient method to deliver hydrophilic polyphenols of salvia miltiorrhiza and carthamus tinctorius for enhanced anti-atherosclerotic effect via metal-phenolic network. Colloids Surf. B Biointerfaces.

